# Massage of the Heart

**Published:** 1908-06-20

**Authors:** 


### Anæsthetics.

### MASSAGE OF THE HEART.

The following case is of more than usual interest, as it demonstrates the value of massage of the heart as an aid in difficult anæsthetic cases. Mrs. C., whose age was said to be 58, had to undergo an operation for an immense ovarian cystoma. On examination it was found that the heart was feeble and irregular, though the sounds were closed. The respiratory and urinary systems were apparently healthy. Ether was given with an ordinary Clover's inhaler, and the patient got well under. The operation was proceeded with, and the cyst was exposed. A large trocar was pushed into the cyst and the fluid contents evacuated. For a few minutes prior to this the patient had been breathing air, the Clover's inhaler having been withdrawn.

When a quantity of the fluid had escaped a sudden change came over the patient. The breathing first became jerky and irregular, and finally stopped. The heart, however, continued to beat for about two minutes after the failure of respiration, but it finally stopped also. Strychnine was injected hypodermically, hot cloths were applied to the præcordia, artificial respiration by Silvester's method was done, and rhythmic traction of the tongue was practised. These efforts were proving apparently of no avail, and the surgeon as a last resort seized the heart through the diaphragm and massaged it. Within a few seconds the heart began to contract, and in a few minutes the cardiac and respiratory functions were again in action.

The patient was given ether by the open method, and the operation continued; but, needless to say, after the alarming interlude this was concluded with all possible speed. The cyst was very adherent, and no attempt was made to remove the posterior part. The free anterior part was ligatured and removed, and the remaining part was brought up and stitched to the abdominal wall. The patient recovered from the anæsthetic, and for a day or so appeared to be doing well. Sloughing of the cyst set in, and she died eleven days after the operation from asthenia.

*Remarks.*—Cases have been from time to time recorded in which heart massage has brought back to life the apparently dead; but in some of them life was maintained for a few hours only. In this case the patient lived for 11 days, and I hardly think the anæsthetic played any part in the fatal issue. No post-mortem was allowed, and therefore one could not examine the heart, which was most probably the seat of fatty degeneration. The patient was very badly preserved, and looked more like 70 than 58.

